# New Ratios for Performance Improvement for Identifying Acyl-CoA Dehydrogenase Deficiencies in Expanded Newborn Screening: A Retrospective Study

**DOI:** 10.3389/fgene.2019.00811

**Published:** 2019-09-18

**Authors:** Benjing Wang, Qin Zhang, Ang Gao, Qi Wang, Jun Ma, Hong Li, Ting Wang

**Affiliations:** ^1^Newborn Screening Laboratory, Center for Reproduction and Genetics, The Affiliated Suzhou Hospital of Nanjing Medical University, Suzhou, China; ^2^Genetic Clinic, Center for Reproduction and Genetics, The Affiliated Suzhou Hospital of Nanjing Medical University, Suzhou, China; ^3^Infertility Clinic, Center for Reproduction and Genetics, The Affiliated Suzhou Hospital of Nanjing Medical University, Suzhou, China

**Keywords:** C4/C5DC+C6-OH, C8/C14:1, C14:1/C16-OH, short chain acyl-CoA dehydrogenase deficiency, medium chain acyl-CoA dehydrogenase deficiency, very long chain acyl-CoA dehydrogenase deficiency, expanded newborn screening

## Abstract

Some success in identifying acyl-CoA dehydrogenase (ACAD) deficiencies before they are symptomatic has been achieved through tandem mass spectrometry. However, there has been several challenges that need to be confronted, including excess false positives, the occasional false negatives and indicators selection. To select ideal indicators and evaluate their performance for identifying ACAD deficiencies, data from 352,119 newborn babies, containing 20 cases, were used in this retrospective study. A total of three new ratios, C4/C5DC+C6-OH, C8/C14:1, and C14:1/C16-OH, were selected from 43 metabolites. Around 903 ratios derived from pairwise combinations of all metabolites *via* multivariate logistic regression analysis were used. In the current study, the regression analysis was performed to identify short chain acyl-CoA dehydrogenase (SCAD) deficiency, medium chain acyl-CoA dehydrogenase (MCAD) deficiency, and very long chain acyl-CoA dehydrogenase (VLCAD) deficiency. In both model-building and testing data, the C4/C5DC+C6-OH, C8/C14:1 and C14:1/C16-OH were found to be better indicators for SCAD, MCAD and VLCAD deficiencies, respectively, compared to [C4, (C4, C4/C2)], [C8, (C6, C8, C8/C2, C4DC+C5-OH/C8:1)], and [C14:1, (C14:1, C14:1/C16, C14:1/C2)], respectively. In addition, 22 mutations, including 5 novel mutations and 17 reported mutations, in *ACADS*, *ACADM*, and *ACADL* genes were detected in 20 infants with ACAD deficiency by using high-thorough sequencing based on target capture. The pathogenic mutations of c.1031A > G in *ACADS*, c.449_452delCTGA in *ACADM* and c.1349G > A in *ACADL* were found to be hot spots in Suzhou patients with SCAD, MCAD, and VLCAD, respectively. In conclusion, we had identified three new ratios that could improve the performance for ACAD deficiencies compared to the used indicators. We considered to utilize C4/C5DC+C6-OH, C8/C14:1, and C14:1/C16-OH as primary indicators for SCAD, MCAD, and VLCAD deficiency, respectively, in further expanded newborn screening practice. In addition, the spectrum of mutations in Suzhou population enriches genetic data of Chinese patients with one of ACAD deficiencies.

## Introduction

Acyl-CoA dehydrogenase (ACAD) deficiencies, are the most common fatty acid oxidation disorders (FAODs), and could be identified by monitoring acyl carnitines (AC) using high performance liquid chromatography tandem mass spectrometry (HPLC-MS/MS) (Gregersen et al., 2001; Yamada and Taketani, 2019). Three acyl-CoA dehydrogenases such as, very long chain acyl-CoA dehydrogenase (VLCAD), medium chain acyl-CoA dehydrogenase (MCAD), and short chain acyl-CoA dehydrogenase (SCAD) are mainly involved in the conversion of very long chain fatty acids to acetyl-CoA. Based on deficiency of specific acyl-CoA dehydrogenase, ACAD deficiencies are classified into VLCAD (OMIM number: 201470), MCAD (OMIM number: 201450), and SCAD deficiency (OMIM number: 609016). Despite ACAD deficiencies always presents as no clinical symptoms in the neonatal period, it can lead to sudden death as a result of a mild, intercurrent illness and fasting (Yusupov et al., 2010; Pryce et al., 2011; Lovera et al., 2012; Baruteau et al., 2013; Yamamoto et al., 2013; Scalais et al., 2015; Yamada and Taketani, 2019). The health damage caused by ACAD deficiencies could be evaded by continuous feeding and paying more attentions during illnesses to diminished dietary intake (Vasiljevski et al., 2018). Fortunately, the introduction of HPLC-MS/MS in newborn screening (NBS) can identify infants with any one of the ACAD deficiencies before they are symptomatic, implement appropriate dietary interventions, and provide them with protocols for emergency situations.

It has been reached at consensus that the conditions of MCAD deficiency and VLCAD deficiency should be included in expanded NBS panels (Dietzen et al., 2009; Burgard et al., 2012). SCAD deficiency are likewise screened by expanded NBS in some regions (Wilcken et al., 2003; Gallant et al., 2012). Despite HPLC-MS/MS use in newborn screening for ACAD deficiencies for over two decades, there has been some challenges that need to be faced, including overabundance cases of false positives (FPs) (Tarini et al., 2006; Tu et al., 2012; Karaceper et al., 2016) and the occasional false negatives (FNs) (Estrella et al., 2014). FPs lead to unnecessary anxiety to the families, additional diagnostic testing, and treatment. FNs can cause a delayed diagnosis and treatment, and poor prognosis. For identifying life-threatening inborn errors of metabolisms (IEMs), it is reasonable to eliminate FNs regardless of the number of FPs. For moderate IEMs, efforts should be made to reduce FPs (Grosse et al., 2010). Due to simultaneous detection of dozens of acylcarnitines by HPLC-MS/MS, one IEM was constantly recognized by observing an essential indicator and several secondary markers. C4, C8, and C14:1 are primary indicators of SCAD, MCAD, and VLCAD deficiency, respectively. All these metabolites are selected to be the primary indicators, because they are the primary substrates of acyl-CoA dehydrogenases. There is no uncertainty that deficiency of these enzymes is associated with accumulation of their substrates. However, due to the complexity of β-oxidation of fatty acids, the levels of these metabolites are affected by very long chain fatty acids, that are the main kind of fatty acids in diet. In addition, secondary indicators used for conditions are not consistent among NBS centers (Han et al., 2007; Lim et al., 2014; Smon et al., 2018);. It was known that, the presence of a small sample size of positive cases, establishing an appropriate cutoff values for multi-indicators is difficult for NBS centers. It seems that excess FPs and the occasional FNs were partially caused by low performance of inappropriate indicators and cutoff values for identifying IEMs.

In China, the utility of HPLC-MS/MS in screening for IEMs was later compared to developed countries, such as the US and the UK. Recently, many newborn screening centers executed the expanded newborn screening program and national accord for screening, diagnosis and treatment for IEMs in the preparation. Nowadays, conditions and their indicators targeted by newborn screening that are listed on a newborn screening website (https://newbornscreeningcodes.nlm.nih.gov) derive from early experience in developed countries. Regardless of whether these conditions and indicators are optimal for Chinese population, is a significant inquiry that needs research *via* analyzing Chinese data. The aim of this present study is to select optimal indicators and evaluate their performance for identifying ACAD deficiencies.

## Materials and Methods

### Study Population

This is a retrospective study, of which the protocol was reviewed and approved by Ethic committee of the affiliated Suzhou hospital of Nanjing Medical University. Of 544,001 newborns born in Suzhou, China, from April 2014 to June 2018, 352,119 (64.7%) cases were referred to expanded newborn screening for inborn metabolic disorders by HPLC-MS/MS (TQD, Waters, USA). Informed and written consent was obtained from the parents of all screened newborns. After 72 h of newborn’s life, three blood spot specimens of each neonates were collected from the heel and spotted on Whatman 903 filter papers (Guthrie card) by skilled nurses. These blood spots were dried at ambient temperature and transported to NBS center at 2–8°C. A 3.2-mm-diameter dried blood spot (DBS) was detached using Puncher 9.0 for HPLC-MS/MS assay.

### HPLC-MS/MS Assay

Using TQD HPLC-MS/MS system (Waters, MA, United States) and NeoBase non-derivatized kit (PerkinElmer, United States), a total of 43 metabolites in a DBS were measured, including 11 amino acids, 30 acyl-carnitines, free carnitine, and succinylacetone. In short, the assay consists of three operations. First, 100 μl extract solution containing internal standards was added into U bottom plates and incubated for 45 min at 45°C. Second, 75 μl extract solution was transferred into V bottom plates. Third, after 2 h stand at ambient temperature, 25 μl solution was used for metabolites analysis on tandem mass spectrometry. Three levels of internal quality controls including blank, low, and high levels of pertinent acylcarnitines in dried blood spot were used.

### Indicators and Positive Criterions for ACAD Deficiencies

The indicators and positive criterions for ACAD deficiencies were shown in **Table 1**. Results containing [C4 > 0.7 nmol/L, or (C4 > 0.5 nmol/L, C4/C2 > 0.03)], [C8 > 0.3 nmol/L, or (C6 > 0.09, C8 > 0.15, C8/C ≥ 0.01, C4DC+C5-OH/C8＜1.3)], [C14:1 > 0.5 or (C14:1 > 0.29, C14:1/C2 ≥ 0.02, C14:1/C16 > 0.1)] are defined as “positive” for SCAD, MCAD, and VLCAD deficiency, respectively. Infants with an initial positive result were recalled for a new specimen. Infants with a second positive result were referred to diagnostic testing and genetic analysis by pediatricians who specialize in the diagnosis and treatment of IEMs.

**Table 1:** Indicators and positive criteria used for ACAD deficiencies in expanded newborn screening.

Conditions	Positive criterion I	Positive criterion II
SCAD deficiency	C4 > 0.7 nmol/L	C4 > 0.5 nmol/L, C4/C2 > 0.03
MCAD deficiency	C8 > 0.3 nmol/L	C6 > 0.09 nmol/L, C8 > 0.15 nmol/L, C8/C2 ≥ 0.01, C4DC+C5-OH/C8 < 1.3
VLCAD deficiency	C14:1 > 0.5 nmol/L	C14:1 > 0.29 nmol/L, C14:1/C2 ≥ 0.02, C14:1/C16 > 0.1

SCAD, short chain acyl-CoA hydrogenase; MCAD, medium chain acyl-CoA hydrogenase; VLCAD, very long chain acyl-CoA hydrogenase.

### Genetic Analysis

High thorough sequencing based on target capture was performed on cases with ACAD deficiencies using the expanded edition panel of IEMs (Genuine Diagnostic, Hangzhou, China) to sequence 306 genes, such as *ACADS* (OMIM number: 606885), *ACADM* (OMIM number: 607008), *ACADL* gene (OMIM number: 609575), and so on. First, the target sequences were enriched based on multiple probe hybridization by using Agilent SureSelect Human Exon Sequence Capture Kit (Agilent Technologies Inc, California, USA). Second, the capture products were purified using Agencourt AMPure XP beads (Beckman Coulter Inc, Miami, USA). Third, the sequencing library was established by using TruePrepTM DNA Library Prep Kit V2 (Vazyme Biotech, New Jersey, USA) and TruePrepTM Index Kit V2 (Vazyme Biotech, New Jersey, USA), and examined by using Agilent High Sensitivity DNA Kit (Agilent Technologies Inc, California, USA). Finally, the sequencing library was quantified by Illumina DNA Standards and Primer Premix Kit (KAPA Biosystems, Boston, USA), and massively parallel sequenced on Illumina HiSeq 2500 system. All mutations were confirmed by using Sanger sequencing.

### Statistical Analysis

Statistical analysis was performed using SPSS17.0 version. To select the optimal indicators, multivariate logistic regression analysis was performed among 946 variates (43 metabolites and 903 ratios derived from all pairwise combinations of 43 metabolites) and ACAD deficiencies. Paired chi-square test was used to compare different PPVs for ACAD deficiencies. Difference of measurement data was compared with analysis of variance. *p* < 0.05 was considered to be statistical significance.

## Results

**Figure 1:**
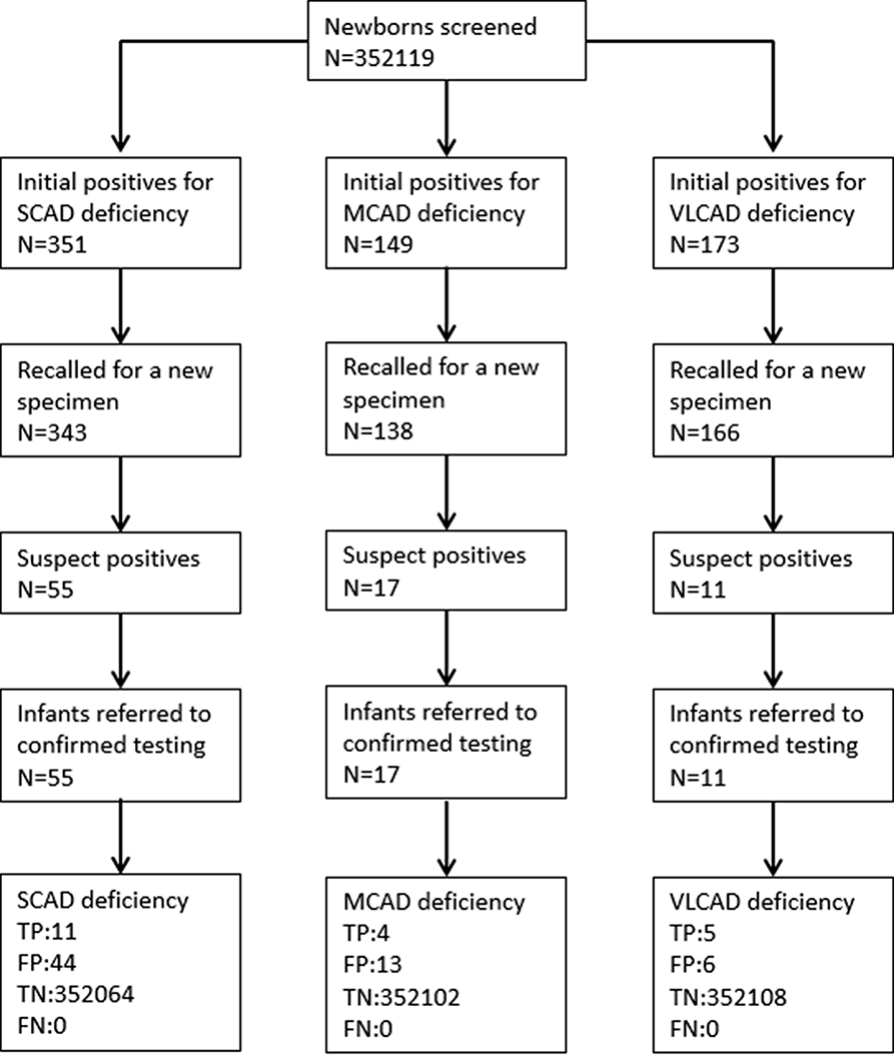
Flowchart of expanded newborn screening for short, medium, and very long chain acyl-CoA dehydrogenase deficiencies. SCAD, short chain acyl-CoA dehydrogenase; MCAD, medium chain acyl-CoA dehydrogenase; VLCAD, very long chain acyl-CoA dehydrogenase; TP, true positive; FP, false positive; TN, true negative; FN, false negative.

A total of 352,119 newborns were tested by HPLC-MS/MS and 20 (1/17,606) infants were confirmed with one of ACAD deficiencies. The characteristics of newborns were shown in **Table 2**. Around 673 (0.19%) newborns had an initial positive result for ACAD deficiencies, of that 647 (96.1%) were recalled for a new specimen. After repeat, 83 (1/4242) newborns were considered as suspect positives of ACAD deficiencies. All infants with suspect positive were further tested by diagnostic testing and genetic analysis. Above all, 11 (1/32,011), 4 (1/88,030), and 5 (1/70,424) infants were diagnosed with SCAD, MCADand VLCAD deficiencies, respectively. The flowchart of newborn screening was presented in **Figure 1**.

**Table 2:** Characteristics of newborns screened by HPLC-tandem mass spectrometry.

	Newborns without acyl-CoA hydrogenase deficiencies N = 352,099	Newborns with false positive result for acyl-CoA hydrogenase deficiency, N = 754	Newborns with acyl-CoA hydrogenase deficiencies N = 20	*p*^#^	P*
Age at initial testing (days, median)	3 (3–20)	3 (3–20)	4 (3–16)	ND	0.085
Gender					
Male	184,917	347	9	0.501	0.563
Female	167,182	326	11		
No record	40	0	0		
Gestational age (weeks)					
<32	1,519	1	0	0.548	0.451
32–36	17,263	28	2		
>37	334,449	638	18		
No record	1,132	6	0		
Birth Weight (g)					
<1,500	559	0	0	0.857	0.813
1,500–1,999	1,741	1	0		
2,000–2,499	7,893	18	1		
>2,500	331,601	650	19		
No record	10,305	4	0		
Number of fetus					
Singleton	349,350	665	19	0.097	0.138
Twins	2,720	8	1		
Triplet	19	0	0		
Register region					
Suzhou	201,259	384	12	0.797	0.793
Others	150,840	289	8		
No record	0	0	0		
Household registration					
Urban	214,647	408	12	0.930	0.955
Rural	137,452	265	8		
No record	0	0	0		

^#^Comparison between group of newborns with acyl-CoA hydrogenase deficiencies and group of newborns without acyl-CoA hydrogenase deficiencies.

*Comparison between group of newborns with acyl-CoA hydrogenase deficiencies and group of newborns with false positive result for acyl-CoA hydrogenase deficiency.

ND, not done.

**Figure 2:**
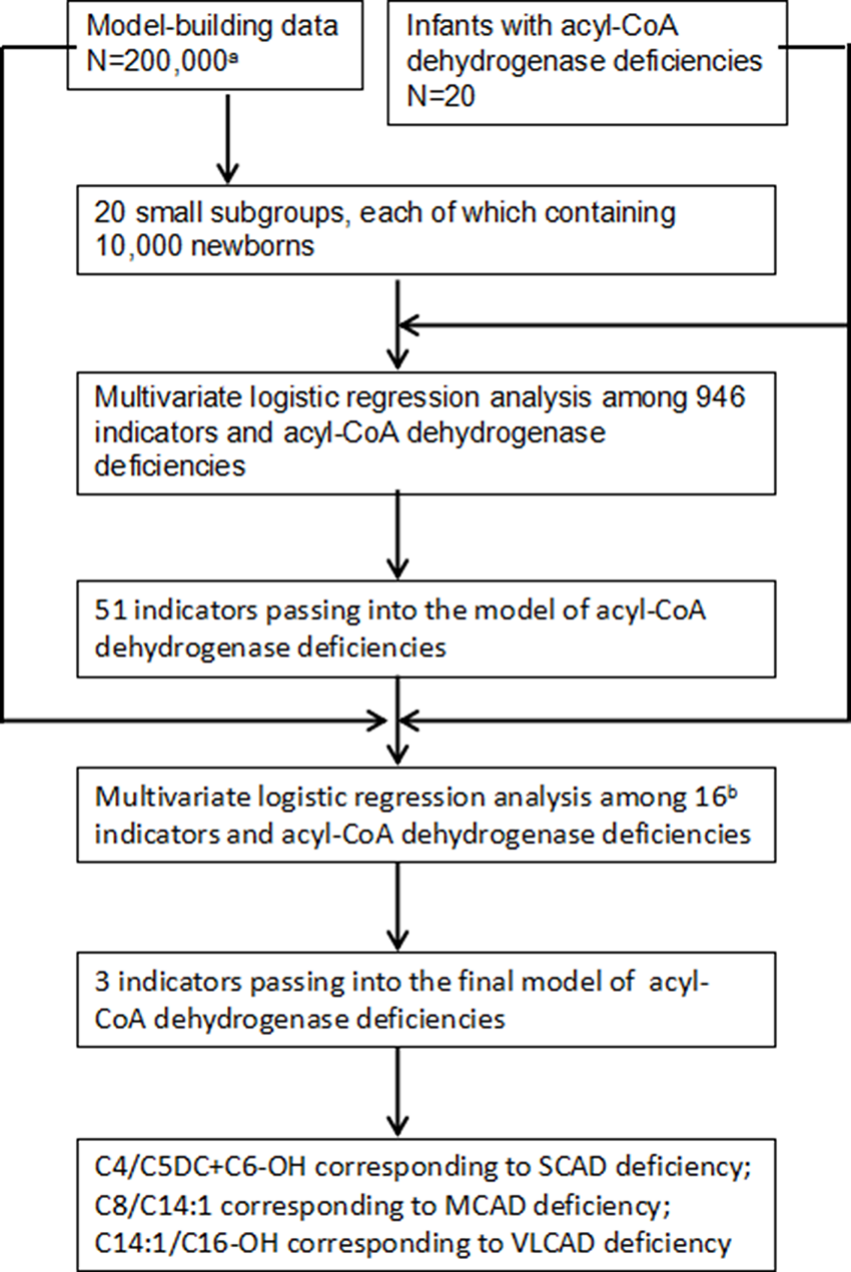
Flowchart of optimal indicators selection. SCAD, short chain acyl-CoA dehydrogenase; MCAD, medium chain acyl-CoA dehydrogenase; VLCAD, very long chain acyl-CoA dehydrogenase. ^a^Newborns screened by tandem mass spectrometry. ^b^Indicators passing into multivariate logistic regression model in at least two small subgroups.

To select optimal indicators, multivariate logistic regression analysis was performed among 946 variates and ACAD deficiencies. Because the data is too large to analyze and calculate at one time using our computer, a protocol of split was designed. The flowchart of marker selection was shown in **Figure 2**. First, all screened newborns were divided into a model-building data (N = 200,000) and a testing data (N = 152,099). The model-building subgroup was further divided into 20 small subgroups, each of which contains 10,000 newborns. Second, in every subgroup, multivariate logistic analysis was performed among the 946 variates and ACAD deficiencies. A total of 51 variates passed into logistic regression models in one or more subgroups (**Supplementary Table 1**), of that 16 passed into model in at least two subgroups. Third, using the model-building data multivariate logistic analysis was performed among those 16 variates and ACAD deficiencies. Only three variates, C4/C5DC+C6-OH, C8/C14:1, and C14:1/C16-OH, passed into the final model of ACAD deficiencies. Finally, these variates were determined as indicators corresponding to SCAD, MCAD, and VLCAD deficiency, respectively.

To evaluate the performance of new ratios using initial screening results in model-building data, the optimal PPV in the condition of 100% sensitivity was compared between used indicators and new ratios (**Table 3**). The optimal PPV of C4/C5DC+C6-OH for SCAD deficiency was 25.0% (11/44), significantly higher than 2.1% (11/523) of C4 (*x*^2^ = 461.65, *p* < 0.001), but not significantly different from 20.8% (11/53) of (C4, C4/C2) (*x*^2^ = 3.000, *p* = 0.083). The optimal PPV of C8/C14:1 for MCAD deficiency was 15.4% (4/26), significantly higher than 0.7% (4/612) of C8 (*x*^2^ = 564.80, *p* < 0.001), and 2.6% (4/153) of (C6, C8, C8/C2, C4DC+C5-OH/C8:1) (*x*^2^ = 102.73, *p* < 0.001). The optimal PPV of C14:1/C16-OH for VLCAD deficiency was 100% (5/5), significantly higher than 19.2% (5/26) of C14:1 (*x*^2^ = 16.00, *p* < 0.001), but not significantly different from 100% (5/5) of (C14:1, C14:1/C16, C14:1/C2).

**Table 3:** Comparison of optimal PPV in the condition of 100% sensitivity for acyl-CoA dehydrogenase deficiencies between new ratios and used indicators using initial screening values in the model-building data.

	TP	FP	*PPV*	*x^2^*	*p*
SCAD					
C4/C5DC+C6-OH	11	33	25.0%	–	–
C4	11	512	2.1%	461.65^a^	<0.001^a^
(C4, C4/C2)	11	42	20.8%	3.00^a^	0.083^a^
MCAD					
C8/C14:1	4	22	18.2%	–	–
C8	4	608	0.7%	564.80^b^	<0.001^b^
(C6, C8, C8/C2, C4DC+C5-OH/C8:1)	4	149	2.6%	102.73^b^	<0.001^b^
VLCAD					
C14:1/C16-OH	5	0	100.0%	–	–
C14:1	5	21	19.2%	16.00^c^	<0.001^c^
(C14:1, C14:1/C16, C14:1/C2)	5	0	100.0%	–^c^	–^c^

SCAD, short chain acyl-CoA hydrogenase; MCAD, medium chain acyl-CoA hydrogenase; VLCAD, very long chain acyl-CoA hydrogenase; TP, true positive; FP, false positive; PPV, positive predictive value.

^a^Compared with C4/C5DC+C6-OH; ^b^compared with C8/C14:1; ^c^compared with C14:1/C16-OH.

Furthermore, using initial screening results, the performance of new ratios in the testing data was compared to that of used indicators for ACAD deficiencies (**Table 4**). The optimal PPV of C4/C5DC+C6-OH for SCAD deficiency was 73.3% (11/15), significantly higher than 3.1% (11/359) of C4 (*x*^2^ = 344.00, *p* < 0.001), and 22.0% (11/50) of (C4, C4/C2) (*x*^2^ = 28.49, *p* < 0.001). The optimal PPV of C8/C14:1 for MCAD deficiency was 18.2% (4/22), significantly higher than 1.0% (4/415) of C8 (*x*^2^ = 372.37, *p* < 0.001), and 3.4% (4/119) of (C6, C8, C8/C2, C4DC+C5-OH/C8:1) (*x*^2^ = 81.00, *p* < 0.001). Similar to in model-building data, in testing data The optimal PPV of C14:1/C16-OH for VLCAD deficiency was 100% (5/5), significantly higher than 38.5% (5/13) of C14:1 (*x*^2^ = 8.00, *p* = 0.005), but not significantly different from 100% (5/5) of (C14:1, C14:1/C16, C14:1/C2).

**Table 4:** Comparison of optimal PPVs in the condition of 100% sensitivity for chain acyl-CoA dehydrogenase deficiencies between new ratio and used indicators using initial screening values in the testing data.

	TP	FP	*PPV*	*x^2^*	*p*
SCAD deficiency					
C4/C5DC+C6-OH	11	4	73.3%	–	–
C4	11	348	3.1%	344.00^a^	<0.001^a^
(C4, C4/C2)	11	39	22.0%	28.49^a^	<0.001^a^
MCAD deficiency					
C8/C14:1	4	18	18.2%	–	–
C8	4	411	1.0%	372.37^b^	<0.001^b^
(C6, C8, C8/C2, C4DC+C5-OH/C8:1)	4	115	3.4%	81.00^b^	<0.001^b^
VLCAD deficiency					
C14:1/C16-OH	5	0	100.0%	–	–
C14:1	5	8	38.5%	8.00^c^	0.005^c^
(C14:1, C14:1/C16, C14:1/C2)	5	0	100.0%	–^c^	–^c^

SCAD, short chain acyl-CoA hydrogenase; MCAD, medium chain acyl-CoA hydrogenase; VLCAD, very long chain acyl-CoA hydrogenase; TP, true positive; FP, false positive; PPV, positive predictive value.

aCompared with C4/C5DC+C6-OH; bcompared with C8/C14:1; ccompared with C14:1/C16-OH.

Clinical characteristics, screening results, and genetic analysis were recorded in **Table 5**. Of 11 infants with SCAD deficiency, 7 (63.6%) are females, and 4 (36.4%) are males. All of 4 infants with MCAD deficiency are males and premature (gestational age = 36^+6^). Of 5 infants with VLCAD deficiency, 4 (80%) are females, and only one (20%) is male, who was born premature and low with birth weight. Except one infant with SCAD deficiency giving up treatment, all were referred to genetic diagnosis. Of the 10 infants with SCAD deficiency, all are homozygous or compound heterozygous for one of eight mutations, including one novel mutation c.1055C > T, and seven reported mutations c.164C > T, c.322G > A, c.737G > A, c.973C > T, c.1031A > G, c.1054G > A, and c.1130C > T, in *ACADS* gene, and eight (80%) carry a pathogenic mutation of c.1031A > G. All infants with SCAD deficiency were referred to control of low fat intake and no fasting, and no one underwent clinical symptoms. At the second screening, five (45.5%) infants with SCAD deficiency had an increased value of C4, but nine (81.8%) infants had an increased value of C4/C5DC+C6-OH. In the present study, four male infants with MCAD deficiency were premature (gestational age < 36^+6^). Further, three (75%) heterozygous infants had five mutations for MCAD deficiency, including two novel mutations c.589A > G and c.1248T > G, and three reported mutations c.449_452delCTGA, c.970G >A, and c.1238G > A in *ACADM* gene, and one (25%) is heterozygous for the novel mutation c.790G > T. Until now, three infants with MCAD deficiency were treated with levocarnitine and referred to control of low fat intake and no fasting, and all of them are asymptomatic. Of five infants with VLCAD deficiency, four (80%) are females, and one (20%) is a male. The male had a gestational age of 34 weeks and a birth weight of 2,450 g. All infants with VLCAD deficiency are homozygous for eight mutations, including two novel mutations c.642_643delCT and c.895C > G, and six reported mutations, c.553G > A, c.848T > C, c.887_888delCT c.1280G > A, c.1345G > C, and c.1349G > A. No one underwent clinical symptoms in the period of treatment with levocarnitine and control of low fat intake and no fasting.

**Table 5:** Clinical characteristics, screening results, and genetic analysis of 20 infants with acyl-CoA hydrogenase deficiency.

No.	Gender	GA(weeks)	BW(g)	Age at initial testing (days)	Initial results	Initial result of new ratios	Age at second testing (days)	Second results	Second results of new ratios	Gene (MIM number)	Mutations (type)	Genetic mode	Treatment	Clinical symptom	Follow up
Infants with SCAD deficiency															
1	Female	38	3,300	16	C4 = 0.89 C4/C2 = 0.18	C4/C5DC+C6-OH = 22.25	27	C4 = 0.91 C4/C2 = 0.09	C4/C5DC+C6-OH = 30.33	*ACADS* (606885)	c.737G > A (Hom)	AR	Low fat intake, no fasting	Normal	Routine
2	Male	38	4,000	3	C4 = 1.42 C4/C2 = 0.06	C4/C5DC+C6-OH = 11.83	9	C4 = 0.8 C4/C2 = 0.1	C4/C5DC+C6-OH = 13.33	*ACADS* (606885)	ND	–	Give up treatment	Unknown	Loss
3	Female	39	3,300	3	C4 = 1.47 C4/C2 = 0.09	C4/C5DC+C6-OH = 12.25	16	C4 = 1.18 C4/C2 = 0.17	C4/C5DC+C6-OH = 19.67	*ACADS* (606885)	c.1031A > G (Het) c.1055C > T (Het)	AR	Low fat intake, no fasting	Normal	Routine
4	Female	38	3,150	12	C4 = 1.08 C4/C2 = 0.14	C4/C5DC+C6-OH = 27.00	21	C4 = 0.98 C4/C2 = 0.12	C4/C5DC+C6-OH = 16.33	*ACADS* (606885)	c.1031A > G (Het) c.1130C > T (Het)	AR	Low fat intake, no fasting	Normal	Routine
5	Female	38	3,050	3	C4 = 2.03 C4/C2 = 0.08	C4/C5DC+C6-OH = 12.69	15	C4 = 1.78 C4/C2 = 0.08	C4/C5DC+C6-OH = 25.43	*ACADS* (606885)	c.1031A > G(Het) c.1054G > A(Het)	AR	Low fat intake, no fasting	Normal	Routine
6	Female	41	4,100	4	C4 = 2.01 C4/C2 = 0.08	C4/C5DC+C6-OH = 12.56	13	C4 = 1.38 C4/C2 = 0.18	C4/C5DC+C6-OH = 15.33	*ACADS* (606885)	c.1031A > G(Het) c.322G > A(Het)	AR	Low fat intake, no fasting	Normal	Routine
7	Male	39	4,100	4	C4 = 1.43 C4/C2 = 0.12	C4/C5DC+C6-OH = 17.88	12	C4 = 1.58 C4/C2 = 0.20	C4/C5DC+C6-OH = 19.75	*ACADS* (606885)	c.164C > T(Het) c.1031A > G(Het)	AR	Low fat intake, no fasting	Normal	Routine
8	Male	37	2,800	3	C4 = 0.51 C4/C2 = 0.04	C4/C5DC+C6-OH = 8.50	10	C4 = 0.54 C4/C2 = 0.06	C4/C5DC+C6-OH = 10.8	*ACADS* (606885)	c.164C > T(Het) c.1130C > T(Het)	AR	Low fat intake, no fasting	Normal	Routine
9	Female	39	3,500	15	C4 = 1.10 ,C4/C2 = 0.13	C4/C5DC+C6-OH = 22.00	23	C4 = 1.30 C4/C2 = 0.17	C4/C5DC+C6-OH = 21.67	*ACADS* (606885)	c.973C > T(Het) c.1031A > G (Het)	AR	Low fat intake, no fasting	Normal	Routine
10	Male	38	3,750	4	C4 = 1.35 C4/C2 = 0.09	C4/C5DC+C6-OH = 12.27	11	C4 = 1.07 C4/C2 = 0.17	C4/C5DC+C6-OH = 15.29	*ACADS* (606885)	c.164C > T (Het) c.1031A > G(Het)	AR	Low fat intake, no fasting	Normal	Routine
11	Female	40	3,650	4	C4 = 0.88 C4/C2 = 0.05	C4/C5DC+C6-OH = 8.80	23	C4 = 1.19 C4/C2 = 0.15	C4/C5DC+C6-OH = 13.22	*ACADS* (606885)	c.1031A > G (Het) c.1130C > T (Het)	AR	Low fat intake, no fasting	Normal	Routine
Infants with MCAD deficiency															
1	Male	37	3,250	3	C6 = 0.92 C8 = 14.52 C8/C2 = 0.7 C4DC+C5-OH/C8 = 0.01	C8/C14:1 = 132.0	17	C6 = 0.68 C8 = 3.44 C8/C2 = 0.42 C4DC+C5-OH/C8 = 0.05	C8/C14:1 = 114.7	*ACADM(607008)*	c.790G > T (Het)	AR	Low fat intake, no fasting, levocarnitine supplementation	Normal	Routine
2	Male	39	3,750	3	C6 = 0.47 C8 = 1.18 C8/C2 = 0.09 C4DC+C5-OH/C8 = 0.13	C8/C14:1 = 29.5	14	C6 = 0.52 C8 = 1.36 C8/C2 = 0.17 C4DC+C5-OH/C8 = 0.18	C8/C14:1 = 34.0	*ACADM(607008)*	c.970G > A(Het) c.1238G > A(Het)	AR	Give up treatment	Unknown	Loss
3	Male	36	2,600	3	C6 = 0.55 C8 = 1.99 C8/C2 = 0.06 C4DC+C5-OH/C8 = 0.08	C8/C14:1 = 15.3	16	C6 = 0.27 C8 = 0.78 C8/C2 = 0.02 C4DC+C5-OH/C8 = 0.17	C8/C14:1 = 4.5	*ACADM(607008)*	c.449_452DelCTGA(Het) c.1248T > G(Het)	AR	Low fat intake, no fasting, levocarnitine supplementation	Normal	Routine
4	Male	39	2,850	3	C6 = 0.09 C8 = 0.17 C8/C2 = 0.01 C4DC+C5-OH/C8 = 1.00	C8/C14:1 = 3.4	11	C6 = 0.13 C8 = 0.22 C8/C2 = 0.02 C4DC+C5-OH/C8 = 0.86	C8/C14:1 = 5.5	*ACADM(607008)*	c.499_452delCTGA(Het) c.589A > G(Het)	AR	Low fat intake, no fasting, levocarnitine supplementation	Normal	Routine
Infants with VLCAD deficiency															
1	Female	37	2,500	9	C14:1 = 1.71 C14:1/C16 = 0.60 C14:1/C2 = 0.51	C14:1/C16-OH = 171.0	18	C14:1 = 1.95 C14:1/C16 = 1.1 C14:1/C2 = 0.78	C14:1/C16-OH = 195.0	*ACADVL(609575)*	c.1280G > A (Het) c.1345G > C (Het)	AR	Low fat intake, no fasting, levocarnitine supplementation	Normal	Routine
2	Female	37	3,500	15	C14:1 = 1.51 C14:1/C16 = 0.62 C14:1/C2 = 0.55	C14:1/C16-OH = 151.0	24	C14:1 = 1.95 C14:1/C16 = 0.71 C14:1/C2 = 0.61	C14:1/C16-OH = 97.5	*ACADVL(609575)*	c.887_888delCT (Hom)	AR	Low fat intake, no fasting, levocarnitine supplementation	Normal	Routine
3	Female	40	3,350	15	C14:1 = 2.1 C14:1/C16 = 1.13 C14:1/C2 = 0.33	C14:1/C16-OH = 210.0	25	C14:1 = 2.91 C14:1/C16 = 1.39 C14:1/C2 = 0.55	C14:1/C16-OH = 145.5	*ACADVL(609575)*	c.642_643delCT(Het) c.1349G > A (Het)	AR	Low fat intake, no fasting, levocarnitine supplementation	Normal	Routine
4	Female	38	3,700	3	C14:1 = 4.27 C14:1/C16 = 0.50 C14:1/C2 = 0.25	C14:1/C16-OH = 61.0	14	C14:1 = 2.91 C14:1/C16 = 1.39 C14:1/C2 = 0.55	C14:1/C16-OH = 170.5	*ACADVL(609575)*	c.1349G > A (Het) c.895C > G (Het)	AR	Low fat intake, no fasting, levocarnitine supplementation	Normal	Routine
5	Male	34	2,450	16	C14:1 = 0.63 C14:1/C16 = 0.53 C14:1/C2 = 0.06	C14:1/C16-OH = 63.0	22	C14:1 = 1.42 C14:1/C16 = 1.15 C14:1/C2 = 0.16	C14:1/C16-OH = 142.0	*ACADVL(609575)*	c.553G > A (Het) c.848T > C(Het)	AR	Low fat intake, no fasting, levocarnitine supplementation	Normal	Routine

SCAD, short chain acyl-CoA hydrogenase; MCAD, medium chain acyl-CoA hydrogenase; VLCAD, very long chain acyl-CoA hydrogenase; ND, not done; AR, autosomal recessive.

## Discussion

Reducing false positives and avoiding false negatives are the most important difficulties that need to be addressed in newborn screening program, especially expanded newborn screening program. Recently, several screening algorithms have been used to improve PPVs and reduce false positives in expanded newborn screening program. Hall et al. (2014a) has improved the laboratory quality by using Region 4 stork (R4), as a postanalytical tool, for VLCAD deficiency by MS/MS. A second-level testing targeting more specific markers and ratios, and genetic testing following screening positives by MS/MS were proposed to decrease false positives and avoid missed diagnosis (Oglesbee et al., 2008; Ficicioglu et al., 2010; Tortorelli et al., 2010; Turgeon et al., 2010; Fisher et al., 2018; Peng et al., 2019);. Recently, evaluation of new ratios derived from pairwise combinations of metabolites measured by expanded newborn screening were conducted to reduce false positives of ACAD deficiencies (Hall et al., 2014b; Merritt et al., 2014; Tajima et al., 2017). In the current study, the used markers had a good performance for identifying SCAD, MCAD, and VLCAD deficiencies. The new ratios showed improved PPVs in the condition of 100% sensitivity in both the model-building data and the testing data. Notably, all the three new ratios for identifying ACAD deficiencies are only associated with metabolites upstream of short chain, medium chain, and very long chain acyl-CoA dehydrogenases.

SCAD deficiency, an autosomal recession disease, is caused by the defect of *ACADS* gene. The reported incidence of SCAD deficiency is 1/25,000–1/50,000, 1/45,000 (Zytkovicz et al., 2001; Loukas et al., 2010; Gallant et al., 2012; Lim et al., 2014). Consistent with previous reports, the incidence of SCAD deficiency is 1/32,011 in Suzhou population. Till date around 70 mutations have been reported in *ACADS* gene, including two common mutations, c.511C > T and c.625G > A (Tonin et al., 2016; Nochi et al., 2017). The remarkable high prevalence of homozygosity for *ACADS* mutations were observed in the general population, with frequencies of approximately 5.5% for c.625G > A and 0.3% for c.511C > T (Nagan et al., 2003; van Maldegem et al., 2005) because most patients with SCAD deficiency are homozygous or compound heterozygous for one of the two common mutations, or they harbor one of them in combination with a rare mutations in *ACADS* gene (Gregersen et al., 1998; Gregersen et al., 2000; Corydon et al., 2001; Gallant et al., 2012; Tonin et al., 2016). Moreover, homozygosity for these mutations is considered to confer susceptibility to clinical diseases (van Maldegem et al., 2010). However, hot-spot mutations vary in different populations. Lisyová et al. (2018) reported c.310_312 del GAG and c.1138C > T were the most common mutations in Slovakia, with allelic frequency of 64% for c.310_312delGAG and 31% for c.1138C > T. Tein and coworkers (2008) observed 100% Ashkenazim patients carried the c.319C > T mutation in *ACADS* gene, including 3 (30%) homozygous for c.319C > T and 7 (70%) compound heterozygous for c.319C > T and c.625G > A. Limited data on the genetic analysis for Chinese patients is available. Huang et al. (2016) found 13 reported mutations of *ACADS* gene in 17 Zhejiang patients. In the latest published study, Guo et al. (2018) found that all the three patients were compound heterozygous for c.164C > T and other mutations that included c.1031A > G, c.770A > G, and c.1064G > A in Jining. Only one (10%) infant is homozygous for a pathogenic mutation c.737G > A, which is a rare mutation in SCAD deficiency. This may be explained by intermarriage. Other mutations identified in Suzhou patients included three reported mutation c.322G > A, c.973C > T, and c.1054G > A, and one novel mutation c.1055C > T. It appears that two mutations c.164C > T and c.1031A > G are the most common mutations in general Chinese patients with SCAD deficiency. As known, almost all patients with SCAD deficiency present no symptoms, though the disease had a wide spectrum of symptoms. In addition, no genotype–phenotype correlation was observed. In Suzhou patients with SCAD deficiency, all of that are asymptomatic in the period of treatment and follow up regardless genotype. No association of genotype with clinical manifestation was also observed, which is consistent with previous reports (Huang et al., 2016; Nochi et al., 2017; Zheng et al., 2017).

Nowadays, C4 followed by a C4/C2 are used as primary and secondary markers, respectively to identify SCAD deficiency in almost all newborn. However, compared to C4 and (C4, C4/C2), C4/C5DC+C6-OH might have a better performance for identifying SCAD deficiency in our study. Till date, research on the efficiency of C4/C5DC+C6-OH as a marker to identify SCAD deficiency is not available. Because C5DC is not identified from C6-OH by tandem mass spectrometry, C5DC+C6-OH is considered as the primary indicator for identifying glutaric aciduria type I (GA I) in expanded newborn screening practice. There is no known metabolic conversion between C5DC and C4. The values of C4 and C4/C2 could be affected by elevated/decreased C6-OH, that could be caused by fatty acids intake. As a result, C4/C5DC+C6-OH had a higher PPV and a lower false positive rate for SCAD deficiency compared to C4 and C4/C2. The association of genotype and C4 concentration was not observed in SCAD deficiency (Jethva and Ficicioglu, 2008; Pedersen et al., 2008; Waisbren et al., 2008; Huang et al., 2016). It appears that C4/C5DC+C6-OH as an optimal indicator for SCAD deficiency is not caused by specific genotype of Suzhou population. We proposed that C4/C5DC+C6-OH may be used as an independent indicator for identifying SCAD deficiency in other populations. This needs further research based on large sample size and multicenters data.

MCAD deficiency, an autosomal recession disease, is caused by the homozygote or compound heterozygote for the pathogenic mutations in *ACADM* gene. MCAD deficiency has a large variation of incidence from 1/10,000 to 1/50,000 (Shibata et al., 2018). Compared to other Asian regions (Shibata et al., 2018), a relatively high incidence of MCAD deficiency in Suzhou is 1/88,030, similar to that in Zhejiang province (Zheng et al., 2017). More than 70 mutations in *ACADM* gene have been found in patients with MCAD deficiency (Andresen et al., 1997; Andresen et al., 2001; Maier et al., 2005). According to previous reports, each nation or ethic group shows a unique spectrum of *ACADM* gene mutations. The c.985A > G was the most common mutation for MCAD deficiency in many countries and regions, including the United Sates (Bentler et al., 2016), Germany (Maier et al., 2005), Denmark (Andresen et al., 2001), Portugal (Ventura et al., 2014; Janeiro et al., 2019), Caucasia (Grosse et al., 2006), and other European countries (Tanaka et al., 1997). However, the c.985A > G is rare in Asian patients with MCAD deficiency. The other most common mutation for MCAD deficiency is c.449_452delCTGA of *ACADM* gene in Asian countries, including Japan (Yokoi et al., 2007; Hara et al., 2016; Tajima et al., 2016), Korea (Ensenauer et al., 2005; Woo et al., 2011), and China (Tong et al., 2019). However, the most common mutation c.449_452delCTGA is considered as a pathogenic or likely pathogenic mutation. Most patients with the mutation allele of c.449_452delCTGA are asymptomatic, and others always present hypoglycemia (Tajima et al., 2016). In our study, 6 *ACADM* mutations were found in 4 infants with MCAD deficiency. Of those mutations, the c.449_452delCTGA, that thoroughly abolishes the activity of MCAD, was found in 2 (50%) cases. Recently, Tong et al. (2019) investigated 12 Zhejiang patients with MCAD deficiency, and found the c.449_452delCTGA was the most common mutation, accounting for 27.3% patients, suggesting a common mutation in Suzhou patients. The c.970G > C heterozygote was detected in a Chinese patient with MCAD deficiency, indicating ethnicity specific mutation (Tong et al., 2019). Zheng et al. (2017) found no association of genotype with clinical manifestation in Zhejiang patients with MCAD deficiency. In Taiwan patients, c.580A > G was observed to be the most common mutation and accounted for two (100%) patients, including one homozygote and one heterozygote, suggesting its common presence in Chinese patients (Chien et al., 2013). The remaining four mutations, including one reported mutation c.1238G > A and three novel mutations c.589A > G, c.790G > T, and c.1248T > G, have not been reported in Chinese patients and other Asian patients, hence acting as a potential candidates for genetic analysis among Chinese patients with MCAD deficiency.

In this present study, we selected indicators *via* multivariate logistic regression analysis, and found C8/C14:1 was the optimal indicator for MCAD deficiency among 43 metabolites and 903 ratios. Unlike the finding of Hall et al. (2014b) that a mild elevation of C8 is partially caused by an isotope effect from C8:1, it appears that a mild elevation of C8 was caused by elevated C14:1. Compared to C8, C8/C14:1 could improve PPV for MCAD deficiency *via* excluding mild elevated C8, that was caused C14:1. Compared to multi-indicator rule for identifying MCAD deficiency, C8/C14:1 is not only able to improve the PPV, but also establish a high efficiency cut off value.

VLCAD deficiency is caused by the defect of the *ACADVL* gene and inherited as autosomal recession mode. VLCAD deficiency has a low incidence, the range of which is 1/380,000–1/1,400,000 in Asian region (Shibata et al., 2018). However, in Suzhou population, the incidence of VLCAD deficiency is 1/70,424, higher than that of Hangzhou province (Zheng et al., 2017) and many Asian countries. A spectrum of more than 400 *ACADVL* gene mutations had been reported so far (www.ncbi.nlm.nih.gov/clinvar/). According to previous reports, the mutational spectrum is very wide in *ACADVL* gene, and only one mutation c.848T > C is considered to be hot spot (Andresen et al., 1999; Miller et al., 2015; Evans et al., 2016; Pena et al., 2016). Evans et al. (2016) found that 43.5% of patients carried the heterozygote for c.848T > C, but no homozygote. Recently, 21 of 46 patients were observed to be homozygous or heterozygous for c.848T > C in a largest sample size studied by Pena et al. (2016). Despite the high prevalence (83.7%) for c.65C > A mutation in Saudi patients with homozygous VLCAD deficiency, it is not considered as a hot spot because this could be caused by a high consanguinity rate (Bayoumi and Yardumian, 2006; Obaid et al., 2018). In Suzhou population, only a case of homozygous c.887_888delCT pathogenic mutation was noted for VLCAD deficiency (Schiff et al., 2013). However, in the same study the remaining 4 (80%) cases are compound heterozygous for one of seven *ACADVL* gene mutations, including two novel mutations c.642_643delCT and c.895C > G, and five reported mutations, c.553G > A, c.848T > C, c.1280G > A, c.1345G > C, and c.1349G > A (Schiff et al., 2013). In our study, only c.1349G > A was detected in two patients, and the others were detected in only one patient. Similarly, in Shanghai patients with VLCAD deficiency, 3 (27.3%) of 11 patients carried a heterozygous for c.1349G > A, that is the most common mutation, whereas none of 11 patients carried the mutation c.887_888delCT (Jinjun et al., 2015). Due to an extreme low incidence of VLCAD deficiency in China, further research based on large sample size and multicenters is needed to evaluate the distribution characteristic of *ACADVL* gene mutations. There is a clear correlation of genotype with phenotype in VLCAD deficiency. For example, the most common mutation c.848T > C was reported to be a mild mutation in several follow up cohorts (Miller et al., 2015; Evans et al., 2016; Pena et al., 2016). This may be caused by c.848T > C that could not absolutely abolish the reactivity of the VLCAD enzyme (Hoffmann et al., 2012). Although the c.65C > A encodes a truncated protein leading to a complete deficiency of the VLCAD enzyme, it only leads to a relatively milder cardiomyopathy phenotype (Obaid et al., 2018).

In newborn screening practice, C14:1 is used as the primary indicator for identifying VLCAD deficiency. As known, long and very long chain fatty acids are the major constituents of fatty acids in diet. As a result, C14:1 is deduced to be affected by nutrient intake, and may lead to false positives and false negatives for VLCAD deficiency. In our study, C14:1/C16-OH has a better PPV for VLCAD deficiency compared to C14:1. Compared to the multi-indicators rule of (C14:1, C14:1/C16, C14:1/C2), C14:1/C16-OH has the same PPV for VLCAD deficiency. Furthermore, we found C14:1/C16 also had a relatively high PPV for VLCAD deficiency (data not shown), identical to that of C14:1/C16-OH. It seems that C14:1/C16-OH or C14:1/C16 should be used as the primary indicator rather than a secondary indicator for VLCAD deficiency.

In addition, in our study a short, medium and very long chain acyl-CoA dehydrogenase deficiency had a sex preference. Whereas, only a few published studies recorded gender information. In Suzhou patients with VLCAD deficiency, females accounted for 80% (4/5) of cases, that is closed to that of Saudi Arabia cases (Obaid et al., 2018), but significantly higher than that of Shanghai cases (Jinjun et al., 2015). Maier et al. (2005)reported that males accounted for 55.7% of patients with MCAD deficiency; however, all Suzhou patients with MCAD deficiency are males. We hypothesize that gender preference in patients with ACAD deficiency might have a variation among regions and populations. Whether the gender preference can be used for performance improvement for identifying AD deficiencies is a question that needs further research.

A little shortage of this study is a small sample size of positive cases. Due to a low incidence of ACAD deficiency, only 20 infants with ACAD deficiency were found in more than 350,000 newborns. Another shortage is only a small part of second screen positives according to new ratios were recalled for a new specimen. As a result, pilot prospective studies are needed to overcome this challenge.

In conclusion, we found three new ratios that were optimal indicators for identifying SCAD, MCAD, and VLCAD deficiency, respectively. These indicators could reduce initial false positives and improve PPVs for identifying ACAD deficiencies without loss of sensitivity. We consider to use the new ratios as primary indicators for ACAD deficiencies in newborn screening. In addition, the spectrum of mutations in Suzhou population enriches genetic data of Chinese patients with ACAD deficiency.

## Data Availability

The datasets for this manuscript are not publicly available because of Privacy Protection. Requests to access the datasets should be directed to , wangbj850113@163.com.

## Ethics Statement

The protocol was reviewed and approved by the ethic committee of the Affiliated Suzhou Hospital of Nanjing Medical University.

## Author Contributions

BW, TW, and HL conceived and designed the research, analyzed data, and wrote the manuscript. BW, QW, and JM conducted the experiments. QZ and AG took part in diagnosis and treatment of infants with IEM. All authors read and approved the manuscript.

## Funding

This study was supported by grants from Suzhou Key Medical Center (SZZX201505), Suzhou Science and Technology Support Program (SYS201649), Jiangsu Maternal and Children Health Care Research Project (F201603 and F201715), Jiangsu Provincial Medical Innovation Team (CXTDB2017013), Suzhou Clinical Medical Expert Team (SZYJTD201708), and Jiangsu Maternal and Children Health Care Key Discipline (FXK201748).

## Conflict of Interest Statement

The authors declare that the research was conducted in the absence of any commercial or financial relationships that could be construed as a potential conflict of interest.

## References

[B1] Andresen B. S. Bross P. Udvari S. Kirk J. Gray G. Kmoch S. (1997). The molecular basis of medium-chain acyl-CoA dehydrogenase (MCAD) deficiency in compound heterozygous patients: is there correlation between genotype and phenotype? Hum. Mol. Genet. 6, 695–707. 10.1093/hmg/6.5.695 9158144

[B2] Andresen B. S. Dobrowolski S. F. O’Reilly L. Muenzer J. McCandless S. E. Frazier D. M. (2001). Medium-chain acyl-CoA dehydrogenase (MCAD) mutations identified by MS/MS-based prospective screening of newborns differ from those observed in patients with clinical symptoms: identification and characterization of a new, prevalent mutation that results in mild MCAD deficiency. Am. J. Hum. Genet. 68, 1408–1418. 10.1086/320602 11349232 PMC1226127

[B3] Andresen B. S. Olpin S. Poorthuis B. J. Scholte H. R. Vianey-Saban C. Wanders R. (1999). Clear correlation of genotype with disease phenotype in very-long-chain acyl-CoA dehydrogenase deficiency. Am. J. Hum. Genet. 64 (2), 479–494. 10.1086/302261 9973285 PMC1377757

[B4] Baruteau J. Sachs P. Broué P. Brivet M. Abdoul H. Vianey-Saban C. (2013). Clinical and biological features at diagnosis in mitochondrial fatty acid beta-oxidation defects: a French pediatric study of 187 patients. J. Inherit. Metab. Dis. 36, 795–803. 10.1007/s10545-012-9542-6 23053472

[B5] Bayoumi R. A. Yardumian A. (2006). Genetic disease in the Arab world. BMJ 333, 819. 10.1136/bmj.39002.350405.80 17053218 PMC1618432

[B6] Bentler K. Zhai S. Elsbecker S. A. Arnold G. L. Burton B. K. Vockley J. (2016). 221 newborn-screened neonates with medium-chain acyl-coenzyme A dehydrogenase deficiency: findings from the Inborn Errors of Metabolism Collaborative. Mol. Genet. Metab. 119, 75–82. 10.1016/j.ymgme.2016.07.002 27477829 PMC5031545

[B7] Burgard P. Rupp K. Lindner M. Haege G. Rigter T. Weinreich S. S. (2012). Newborn screening programmes in Europe; arguments and efforts regarding harmonization. Part 2. From screening laboratory results to treatment, follow-up and quality assurance. J. Inherit. Metab. Dis. 35, 613–625. 10.1007/s10545-012-9484-z 22544437

[B8] Chien Y. H. Lee N. C. Chao M. C. Chen L. C. Chen L. H. Chien C. C. (2013). Fatty Acid oxidation disorders in a Chinese population in Taiwan. JIMD Rep. 11, 165–172. 10.1007/8904_2013_236 23700290 PMC3755561

[B9] Corydon M. J. Vockley J. Rinaldo P. Rhead W. J. Kjeldsen M. Winter V. (2001). Role of common gene variations in the molecular pathogenesis of short-chain acyl-CoA dehydrogenase deficiency. Pediatr. Res. 49, 18–23. 10.1203/00006450-200101000-00008 11134486

[B10] Dietzen D. J. Rinaldo P. Whitley R. J. Rhead W. J. Hannon W. H. Garg U. C. (2009). National academy of clinical biochemistry laboratory medicine practice guidelines: follow-up testing for metabolic disease identified by expanded newborn screening using tandem mass spectrometry; executive summary. Clin. Chem. 55, 1615–1626. 10.1373/clinchem.2009.131300 19574465

[B11] Ensenauer R. Winters J. L. Parton P. A. Kronn D. F. Kim J. W. Matern D. (2005). Genotypic differences of MCAD deficiency in the Asian population: novel genotype and clinical symptoms preceding newborn screening notification. Genet. Med. 7, 339–343. 10.1097/01.GIM.0000164548.54482.9D 15915086

[B12] Estrella J. Wilcken B. Carpenter K. Bhattacharya K. Tchan M. Wiley V. (2014). Expanded newborn screening in New South Wales: missed cases. J. Inherit. Metab. Dis. 37, 881–887. 10.1007/s10545-014-9727-2 24970580

[B13] Evans M. Andresen B. S. Nation J. Boneh A. (2016). VLCAD deficiency: follow-up and outcome of patients diagnosed through newborn screening in Victoria. Mol. Genet. Metab. 118, 282–287. 10.1016/j.ymgme.2016.05.012 27246109

[B14] Ficicioglu C. Coughlin C. R. Bennett M. J. Yudkoff M. (2010). Very long-chain acyl-CoA dehydrogenase deficiency in a patient with normal newborn screening by tandem mass spectrometry. J. Pediatr. 156, 492–494. 10.1016/j.jpeds.2009.10.031 20056241

[B15] Fisher L. Davies C. Al-Dirbashi O. Y. Ten Brink H. J. Chakraborty P. Lepage N. (2018). A novel method for quantitation of acylglycines in human dried blood spots by UPLC-tandem mass spectrometry. Clin. Biochem. 54, 131–138. 10.1016/j.clinbiochem.2018.01.020 29402417

[B16] Gallant N. M. Leydiker K. Tang H. Feuchtbaum L. Lorey F. Puckett R. (2012). Biochemical, molecular, and clinical characteristics of children with short chain acyl-CoA dehydrogenase deficiency detected by newborn screening in California. Mol. Genet. Metab. 106, 55–61. 10.1016/j.ymgme.2012.02.007 22424739

[B17] Gregersen N. Andresen B. S. Bross P. (2000). Prevalent mutations in fatty acid oxidation disorders: diagnostic considerations.Eur. J. Pediatr. 159, S213–S218. 10.1007/PL00014406 11216903

[B18] Gregersen N. Andresen B. S. Corydon M. J. Corydon T. J. Olsen R. K. Bolund L. (2001). Mutation analysis in mitochondrial fatty acid oxidation defects: exemplified by acyl-CoA dehydrogenase deficiencies, with special focus on genotype-phenotype relationship. Hum. Mutat. 18, 169–189. 10.1002/humu.1174 11524729

[B19] Gregersen N. Winter V. S. Corydon M. J. Corydon T. J. Rinaldo P. Ribes A. (1998). Identification of four new mutations in the short-chain acyl-CoAdehydrogenase (SCAD) gene in two patients: one of the variant alleles, 511C–>T, is present at an unexpectedly high frequency in the general population, as was the case for 625G–>A, together conferring susceptibility to ethylmalonicaciduria. Hum. Mol. Genet. 7, 619–627. 10.1093/hmg/7.4.619 9499414

[B20] Grosse S. D. Khoury M. J. Greene C. L. Crider K. S. Pollitt R. J. (2006). The epidemiology of medium chain acyl-CoA dehydrogenase deficiency: an update. Genet. Med. 8, 205–212. 10.1097/01.gim.0000204472.25153.8d 16617240

[B21] Grosse S. D. Rogowski W. H. Ross L. F. Cornel M. C. Dondorp W. J. Khoury M. J. (2010). Population screening for genetic disorders in the 21st century: evidence, economics, and ethics. Public Health Genomics 13, 106–115. 10.1159/000226594 19556749

[B22] Guo K. Zhou X. Chen X. Wu Y. Liu C. Kong Q. (2018). Expanded Newborn screening for inborn errors of metabolism and genetic characteristics in a chinese population. Front. Genet. 9, 122. 10.3389/fgene.2018.00122 29731766 PMC5920142

[B23] Hall P. L. Marquardt G. McHugh D. M. Currier R. J. Tang H. Stoway S. D. (2014a). Postanalytical tools improve performance of newborn screening by tandem mass spectrometry. Genet. Med. 16, 889–895. 10.1038/gim.2014.62 24875301 PMC4262759

[B24] Hall P. L. Wittenauer A. Hagar A. (2014b). Newborn screening for medium chain acyl-CoA dehydrogenase deficiency: performance improvement by monitoring a new ratio. Mol. Genet. Metab. 113, 274–277. 10.1016/j.ymgme.2014.10.007 25454677

[B25] Hara K. Tajima G. Okada S. Tsumura M. Kagawa R. Shirao K. (2016). Significance of ACADM mutations identified through newborn screening of MCAD deficiency in Japan. Mol. Genet. Metab. 118, 9–14. 10.1016/j.ymgme.2015.12.011 26947917

[B26] Han L. S. Ye J. Qiu W. J. Gao X. L. Wang Y. Gu X. F. (2007). Selective screening for inborn errors of metabolism on clinical patients using tandem mass spectrometry in China: a four-year report. J. Inherit. Metab. Dis. 30, 507–514. 10.1007/s10545-007-0543-9 17347912

[B27] Hoffmann L. Haussmann U. Mueller M. Spiekerkoetter U. (2012). VLCAD enzyme activity determinations in newborns identified by screening: a valuable tool for risk assessment. J. Inherit. Metab. Dis. 35, 269–277. 10.1007/s10545-011-9391-8 21932095

[B28] Huang X. W. Zhang Y. Yang J. B. Hong F. Qian G. L. Tong F. (2016). [Clinical, biochemical and gene mutation characteristics of short chain acyl-coenzyme A dehydrogenase deficiency by neonatal screening]. Zhonghua Er Ke Za Zhi 54, 927–930. 10.3760/cma.j.issn.0578-1310.2016.12.011 27938594

[B29] Janeiro P. Jotta R. Ramos R. Florindo C. Ventura F. V. Vilarinho L. (2019). Follow-up of fatty acid β-oxidation disorders in expanded newborn screening era. Eur. J. Pediatr. 178, 387–394. 10.1007/s00431-018-03315-2 30617651

[B30] Jethva R. Ficicioglu C. (2008). Clinical outcomes of infants with short-chain acyl-coenzyme A dehydrogenase deficiency (SCADD) detected by newborn pscreening. Mol. Genet. Metab. 95, 241–242. 10.1016/j.ymgme.2008.09.003 18951053

[B31] Jinjun C. Wenjuan Q. Ruinan Z. Jun Y. Lianshu H. Huiwen Z. (2015). [Clinical features and ACADVL gene mutation spectrum analysis of 11 Chinese patients with very long chain acyl-CoA dehydrogenase deficiency]. Zhonghua Er Ke Za Zhi 53, 262–267. 10.3760/cma.j.issn.0578-1310.2015.04.007 26182500

[B32] Karaceper M. D. Chakraborty P. Coyle D. Wilson K. Kronick J. B. Hawken S. (2016). The health system impact of false positive newborn screening results for medium-chain acyl-CoA dehydrogenase deficiency: a cohort study. Orphanet. J. Rare Dis. 11, 12. 10.1186/s13023-016-0391-5 26841949 PMC4741015

[B33] Lim J. S. Tan E. S. John C. M. Poh S. Yeo S. J. Ang J. S. (2014). Inborn Error of Metabolism (IEM) screening in Singapore by electrospray ionization-tandem mass spectrometry (ESI/MS/MS): an 8 year journey from pilot to current program. Mol. Genet. Metab. 113, 53–61. 10.1016/j.ymgme.2014.07.018 25102806

[B34] Lisyová J. Chandoga J. Jungová P. Repiský M. Knapková M. Machková M. (2018). An unusually high frequency of SCAD deficiency caused by two pathogenic variants in the ACADS gene and its relationship to the ethnic structure in Slovakia.BMC. Med. Genet. 19, 64. 10.1186/s12881-018-0566-0 PMC5910552 29678161

[B35] Lovera C. Porta F. Caciotti A. Catarzi S. Cassanello M. Caruso U. (2012). Sudden unexpected infant death (SUDI) in a newborn due to medium chain acyl CoA dehydrogenase (MCAD) deficiency with an unusual severe genotype. Ital. J. Pediatr. 38, 59. 10.1186/1824-7288-38-59 23095120 PMC3502270

[B36] Loukas Y. L. Soumelas G. S. Dotsikas Y. Georgiou V. Molou E. Thodi G. (2010). Expanded newborn screening in Greece: 30 months of experience. J. Inherit. Metab. Dis. 33 (Suppl. 3), S341–S348. 10.1007/s10545-010-9181-8 20721692

[B37] Maier E. M. Liebl B. Röschinger W. Nennstiel-Ratzel U. Fingerhut R. Olgemöller B. (2005). Population spectrum of ACADM genotypes correlated to biochemical phenotypes in newborn screening for medium-chain acyl-CoA dehydrogenase deficiency. Hum. Mutat. 25, 443–452. 10.1002/humu.20163 15832312

[B38] Merritt J. L. Vedal S. Abdenur J. E. Au S. M. Barshop B. A. Feuchtbaum L. (2014). Infants suspected to have very-long chain acyl-CoA dehydrogenase deficiency from newborn screening. Mol. Genet. Metab. 111, 484–492. 10.1016/j.ymgme.2014.01.009 24503138

[B39] Miller M. J. Burrage L. C. Gibson J. B. Strenk M. E. Lose E. J. Bick D. P. (2015). Recurrent ACADVL molecular findings in individuals with a positive newborn screen for very long chain acyl-coA dehydrogenase (VLCAD) deficiency in the United States. Mol. Genet. Metab. 116, 139–145. 10.1016/j.ymgme.2015.08.011 26385305 PMC4790081

[B40] Nagan N. Kruckeberg K. E. Tauscher A. L. Bailey K. S. Rinaldo P. Matern D. (2003). The frequency of short-chain acyl-CoA dehydrogenase gene variants in the US population and correlation with the C(4)-acylcarnitine concentration in newborn blood spots. Mol. Genet. Metab. 78, 239–246. 10.1016/S1096-7192(03)00034-9 12706374

[B41] Nochi Z. Olsen R. K. J. Gregersen N. (2017). Short-chain acyl-CoA dehydrogenase deficiency: from gene to cell pathology and possible disease mechanisms. J. Inherit. Metab. Dis. 40, 641–655. 10.1007/s10545-017-0047-1 28516284

[B42] Obaid A. Nashabat M. Alfadhel M. Alasmari A. Al Mutairi F. Alswaid A. (2018). Clinical, Biochemical, and Molecular Features in 37 Saudi Patients with Very Long Chain Acyl CoA Dehydrogenase Deficiency. JIMD Rep. 40, 47–53. 10.1007/8904_2017_58 28980192 PMC6122013

[B43] Oglesbee D. Sanders K. A. Lacey J. M. Magera M. J. Casetta B. Strauss K. A. (2008). Second-tier test for quantification of alloisoleucine and branched-chain amino acidsin dried blood spots to improve newborn screening for maple syrup urine disease(MSUD). Clin. Chem. 54, 542–549. 10.1373/clinchem.2007.098434 18178665

[B44] Pedersen C. B. Kølvraa S. Kølvraa A. Stenbroen V. Kjeldsen M. Ensenauer R. (2008). The ACADS gene variation spectrum in 114 patients with short-chain acyl-CoA dehydrogenase (SCAD) deficiency is dominated by missense variations leading to protein misfolding at the cellular level. Hum. Genet. 124, 43–56. 10.1007/s00439-008-0521-9 18523805

[B45] Pena L. D. van Calcar S. C. Hansen J. Edick M. J. Walsh Vockley C. Leslie N. (2016). Outcomes and genotype-phenotype correlations in 52 individuals with VLCAD deficiency diagnosed by NBS and enrolled in the IBEM-IS database. Mol. Genet. Metab. 118, 272–281. 10.1016/j.ymgme.2016.05.007 27209629 PMC4970910

[B46] Peng G. Shen P. Gandotra N. Le A. Fung E. Jelliffe-Pawlowski L. (2019). Combining newborn metabolic and DNA analysis for second-tier testing of methylmalonic acidemia. Genet. Med. 21, 896–903. 10.1038/s41436-018-0272-5 30209273 PMC6416784

[B47] Pryce J. W. Weber M. A. Heales S. Malone M. Sebire N. J. (2011). Tandem mass spectrometry findings at autopsy for detection of metabolic disease in infant deaths: postmortem changes and confounding factors. J. Clin. Pathol. 64, 1005–1009. 10.1136/jclinpath-2011-200218 21896576

[B48] Scalais E. Bottu J. Wanders R. J. Ferdinandusse S. Waterham H. R. De Meirleir L. (2015). Familial very long chain acyl-CoA dehydrogenase deficiency as a cause of neonatal sudden infant death: improved survival by prompt diagnosis. Am. J. Med. Genet. A 167A, 211–214. 10.1002/ajmg.a.36803 25338548

[B49] Schiff M. Mohsen A. W. Karunanidhi A. McCracken E. Yeasted R. Vockley J. (2013). Molecular and cellular pathology of very-long-chain acyl-CoA dehydrogenase deficiency. Mol. Genet. Metab. 109, 21–27. 10.1016/j.ymgme.2013.02.002 23480858 PMC3628282

[B50] Shibata N. Hasegawa Y. Yamada K. Kobayashi H. Purevsuren J. Yang Y. (2018). Diversity in the incidence and spectrum of organic acidemias, fatty acid oxidation disorders, and amino acid disorders in Asian countries: selective screening vs. Expanded newborn screening. Mol. Genet. Metab. Rep. 16, 5–10. 10.1016/j.ymgmr.2018.05.003 29946514 PMC6014585

[B51] Smon A. Repic Lampret B. Groselj U. Zerjav Tansek M. Kovac J. Perko D. (2018). Next generation sequencing as a follow-up test in an expanded newborn screening programme. Clin. Biochem. 52, 48–55. 10.1016/j.clinbiochem.2017.10.016 29111448

[B52] Tajima G. Hara K. Tsumura M. Kagawa R. Okada S. Sakura N. (2016). Screening of MCAD deficiency in Japan: 16years’ experience of enzymatic and genetic evaluation. Mol. Genet. Metab. 119, 322–328. 10.1016/j.ymgme.2016.10.007 27856190

[B53] Tajima G. Hara K. Tsumura M. Kagawa R. Okada S. Sakura N. (2017). Newborn screening for carnitine palmitoyltransferase II deficiency using (C16+C18:1)/C2: evaluation of additional indices for adequate sensitivity and lower false-positivity. Mol. Genet. Metab. 122, 67–75. 10.1016/j.ymgme.2017.07.011 28801073

[B54] Tanaka K. Gregersen N. Ribes A. Kim J. Kølvraa S. Winter V. (1997). A survey of the newborn populations in Belgium, Germany, Poland, CzechRepublic, Hungary, Bulgaria, Spain, Turkey, and Japan for the G985 variant allele with haplotype analysis at the medium chain Acyl-CoA dehydrogenasegene locus: clinical and evolutionary consideration. Pediatr. Res. 41, 201–209. 10.1203/00006450-199702000-00008 9029639

[B55] Tarini B. A. Christakis D. A. Welch H. G. (2006). State newborn screening in the tandem mass spectrometry era: more tests, more false-positive results. Pediatrics 118, 448–456. 10.1542/peds.2005-2026 16882794

[B56] Tein I. Elpeleg O. Ben-Zeev B. Korman S. H. Lossos A. Lev D. (2008). Short-chain acyl-CoA dehydrogenase gene mutation (c.319C>T) presents with clinical heterogeneity and is candidate founder mutation in individuals of Ashkenazi Jewish origin. Mol. Genet. Metab. 93, 179–189. 10.1016/j.ymgme.2007.09.021 18054510

[B57] Tong F. Jiang P. P. Yang R. L. Huang X. L. Zhou X. L. Hong F. (2019). [Medium-chain acyl-CoA dehydrogenase deficiency: neonatal screening and follow-uP]. Zhongguo Dang Dai Er Ke Za Zhi 21, 52–57. 10.7499/j.issn.1008-8830.2019.01.010 30675864 PMC7390178

[B58] Tonin R. Caciotti A. Funghini S. Pasquini E. Mooney S. D. Cai B. (2016). Clinical relevance of short-chain acyl-CoA dehydrogenase (SCAD) deficiency: exploring the role of new variants including the first SCAD-disease-causing allele carrying a synonymous mutation. BBA Clin. 5, 114–119. 10.1016/j.bbacli.2016.03.004 27051597 PMC4816031

[B59] Tortorelli S. Turgeon C. T. Lim J. S. Baumgart S. Day-Salvatore D. L. Abdenur J. (2010). Two-tier approach to the newborn screening of methylenetetrahydrofolate reductase deficiency and other remethylation disorders with tandem mass spectrometry. J. Pediatr. 157, 271–275. 10.1016/j.jpeds.2010.02.027 20394947

[B60] Tu W. J. He J. Chen H. Shi X. D. Li Y. (2012). Psychological effects of false-positive results in expanded newborn screening in China. PLoS One 7, e36235. 10.1371/journal.pone.0036235 22558398 PMC3338668

[B61] Turgeon C. T. Magera M. J. Cuthbert C. D. Loken P. R. Gavrilov D. K. Tortorelli S. (2010). Determination of total homocysteine, methylmalonic acid, and 2-methylcitric acid in dried blood spots by tandem mass spectrometry. Clin. Chem. 56, 1686–1695. 10.1373/clinchem.2010.148957 20807894

[B62] van Maldegem B. T. Wanders R. J. Wijburg F. A. (2010). Clinical aspects of short-chain acyl-CoA dehydrogenase deficiency. J. Inherit. Metab. Dis. 33, 507–511. 10.1007/s10545-010-9080-z 20429031 PMC2946545

[B63] van Maldegem B. T. Waterham H. R. Duran M. van der Vlies M. van Woerden C. S. Bobu L. (2005). The 625G>A SCAD gene variant is common but not associated with increased C4-carnitine in newborn blood spots. J. Inherit. Metab. Dis. 28, 557–562. 10.1007/s10545-005-0557-0 15902559

[B64] Vasiljevski E. R. Summers M. A. Little D. G. Schindeler A. (2018). Lipid storage myopathies: current treatments and future directions. Prog. Lipid Res. 72, 1–17. 10.1016/j.plipres.2018.08.001 30099045

[B65] Ventura F. V. Leandro P. Luz A. Rivera I. A. Silva M. F. Ramos R. (2014). Retrospective study of the medium-chain acyl-CoA dehydrogenase deficiency in Portugal. Clin. Genet. 85, 555–561. 10.1111/cge.12227 23829193

[B66] Waisbren S. E. Levy H. L. Noble M. Matern D. Gregersen N. Pasley K. (2008). Short-chain acyl-CoA dehydrogenase (SCAD) deficiency: an examination of the medical and neurodevelopmental characteristics of 14 cases identified through newborn screening or clinical symptoms. Mol. Genet. Metab. 95, 39–45. 10.1016/j.ymgme.2008.06.002 18676165 PMC4204643

[B67] Wilcken B. Wiley V. Hammond J. Carpenter K. (2003). Screening newborns for inborn errors of metabolism by tandem mass spectrometry. N. Engl. J. Med. 348, 2304–2312. 10.1056/NEJMoa025225 12788994

[B68] Woo H. I. Park H. D. Lee Y. W. Lee D. H. Ki C. S. Lee S. Y. (2011). Clinical, biochemical and genetic analyses in two Korean patients with medium-chain acyl-CoA dehydrogenase deficiency. Korean J. Lab. Med. 31, 54–60. 10.3343/kjlm.2011.31.1.54 21239873 PMC3111034

[B69] Yamada K. Taketani T. (2019). Management and diagnosis of mitochondrial fatty acid oxidation disorders: focus on very-long-chain acyl-CoA dehydrogenase deficiency. J. Hum. Genet. 64, 73–85. 10.1038/s10038-018-0527-7 30401918

[B70] Yamamoto A. Nakamura K. Matsumoto S. Iwai M. Shigematsu Y. Tajima G. (2013). VLCAD deficiency in a patient who recovered from ventricular fibrillation, but died suddenly of a respiratory syncytial virus infection. Pediatr. Int. 55, 775–778. 10.1111/ped.12111 24330285

[B71] Yokoi K. Ito T. Maeda Y. Nakajima Y. Ueta A. Nomura T. (2007). Acylcarnitine profiles during carnitine loading and fasting tests in a Japanese patient with medium-chain acyl-CoA dehydrogenase deficiency. Tohoku J. Exp. Med. 213, 351–359. 10.1620/tjem.213.351 18075239

[B72] Yusupov R. Finegold D. N. Naylor E. W. Sahai I. Waisbren S. Levy H. L. (2010). Sudden death in medium chain acyl-coenzyme a dehydrogenase deficiency (MCADD) despite newborn screening. Mol. Genet. Metab. 101, 33–39. 10.1016/j.ymgme.2010.05.007 20580581

[B73] Zheng J. Zhang Y. Hong F. Yang J. Tong F. Mao H. (2017). [Screening for fatty acid oxidation disorders of newborns in Zhejiang province: prevalence, outcome and follow-up]. Zhejiang Da Xue Xue Bao Yi Xue Ban 46, 248–255. 10.3785/j.issn.1008-9292.2017.06.04 29039165 PMC10397024

[B74] Zytkovicz T. H. Fitzgerald E. F. Marsden D. Larson C. A. Shih V. E. Johnson D. M. (2001). Tandem mass spectrometric analysis for amino, organic, and fatty acid disorders in newborn dried blood spots: a two-year summary from the New England Newborn Screening Program. Clin. Chem. 47, 1945–1955. 11673361

